# Dental and general injuries among ski and snowboard instructors in Switzerland, Germany, and Austria–A questionnaire‐based study

**DOI:** 10.1002/cre2.518

**Published:** 2021-12-14

**Authors:** Greta Unzeitig, Florin Eggmann, Andreas Filippi

**Affiliations:** ^1^ Department of Oral Surgery and Center of Dental Traumatology University Center for Dental Medicine Basel UZB, University of Basel Basel Switzerland; ^2^ Department of Periodontology, Endodontology and Cariology University Center for Dental Medicine UZB, University of Basel Basel Switzerland; ^3^ Present address: Greta Unzeitig Runzstraße 44 Freiburg Germany

**Keywords:** dental first aid know‐how, helmet, mouthguard, snow sport injuries, tooth rescue box

## Abstract

**Objectives:**

Data on the injury rate of skiers and snowboarders are currently limited. The aim of this study was, therefore, to assess the frequency of general and dental injuries among snow sports instructors, to investigate the use of protective gear and mouthguards, and to evaluate snow sports instructors' dental first aid know‐how.

**Material and Methods:**

A questionnaire‐based, cross‐sectional study comprising 603 ski and snowboard instructors from Austria, Germany, and Switzerland was conducted in the timeframe December 2019 to May 2020. The survey gathered data on general and dental injuries sustained by instructors, protective gear usage, and know‐how in dental first aid. The statistical analysis included *χ* ^2^ tests, Wilcoxon rank‐sum and Kruskal–Wallis tests, and linear regression analysis. The level of significance was set at *α* = .05.

**Results:**

Out of the 603 instructors, 326 (54.1%) sustained an injury while skiing or snowboarding. Forty (6.6%) reported a snow sports‐related dental injury. The rates of injuries related to skiing and snowboarding showed no significant difference (*p* = .0952). Compared with snowboarding on slopes, backcountry snowboarding entailed fewer risks of injury for snowboard instructors (*p* = .012). Knowledge of dental first aid was limited, with 45.8% of instructors uninformed about the possibility of replanting avulsed teeth. 10.1% of instructors were familiar with tooth rescue boxes. None of the instructors surveyed had a tooth rescue box in their first aid equipment. Helmet usage was high (95.6%) among snow sports instructors, whereas mouthguard usage was rare (3.5%).

**Conclusions:**

Protective gear usage among snow sports instructors is high. The risk of dental injury while skiing or snowboarding is lower compared with other sports. Dental first aid know‐how ought to be enhanced in snow sports communities to ensure that appropriate first aid is provided in case of a dental injury related to skiing or snowboarding.

## 1 INTRODUCTION

Skiing and snowboarding, although considered an extreme sport by some researchers (Laver et al., 2017), entail fewer risks of sustaining general injuries compared with other popular sports such as soccer, ice hockey, and rugby (Johnson et al., 2008). The injury rate for skiers is estimated at 0.6 injuries per 1000 skier days (Ruedl et al., 2014). The injury rate has shown a steady decrease over recent years, with some studies reporting a 55% drop of injury rates in the period from 1972 to 2006 (Johnson et al., 2008; Ruedl et al., 2014). Nevertheless, owing to the popularity of snow sports, the burden of injuries remains a serious challenge for injury prevention. Injuries of skiers and snowboarders are most frequently caused by individual accidents (87%) rather than collisions with other skiers or snowboarders, (8%); (Ruedl et al., 2014). The most common injuries of skiers and snowboarders, ranging from relatively minor to severe, show distinct differences: wrist injuries, shoulder soft tissue injuries, ankle injuries, concussions, and clavicle fractures are common among snowboarders, whereas skiers more frequently incur anterior collateral and medial collateral ligament injuries, lower extremity contusions, and tibia fractures (Kim et al., 2012). Dentoalveolar injuries occur in almost half of the patients who sustain a skiing‐related facial injury (Gassner et al., 2000). In the management of tooth avulsion, which is one of the most severe dentoalveolar injuries, it is of paramount importance to take appropriate first aid measures immediately after an accident. The treatment costs for the management of an avulsion injury are estimated to range between 5000 and $20,000 over a lifetime (Young et al., 2015). Adequate first aid provided by instructors, can render prosthodontic treatment unnecessary and thus prevent high follow‐up costs. Tooth rescue boxes (SOS Zahnbox, Fa. Hager & Werken, Duisburg; Dentosafe, Fa. Medice Arzneimittel Pütter, Iserlohn), available in pharmacies in Austria, Germany, and Switzerland, are easy to use for laypeople and provide excellent storage conditions for avulsed teeth, thus significantly increasing the chances of a favorable outcome after replantation in a dental setting (Filippi et al., 2008).

To ensure the provision of appropriate first aid, straightforward, evidence‐based measures must be effectively disseminated in snow sports communities. Ski and snowboard instructors are an important target group to support the endeavor to prevent and reduce injuries including dentoalveolar traumas. The aim of this study was, therefore, to comprehensively assess the frequency of general and dental injuries among ski and snowboard instructors and their students, to investigate the use of protective gear and mouthguards, and to evaluate their dental first aid know‐how.

## 2 MATERIALS AND METHODS

The cross‐sectional study was approved by the local ethics committee (EKNZ Req‐2019‐01179). All participating ski and snowboard instructors gave their informed consent to use their anonymized survey data for the purpose of this study. The questionnaire was adapted from validated questionnaires used in other studies on sports‐related general and dental injuries and their prevention (Gass et al., 2016; Innerhofer et al., 2013; Müller et al., 2008). The questionnaire reported in detail in the appendix, comprised 24 items: 4 open‐ended questions and 20 single or multiple‐choice questions. Three questionnaire items (Item 1–3) assessed demographic data, two items (Item 4–5) focused on the instructors' professional training and occupation, eight items (Item 6–13) included questions about snow sports‐related injuries sustained by the instructors themselves, one item (Item 14) concerned snow sports‐related injuries sustained by instructors' students, seven items (Item 15–21) evaluated the instructors' dental first aid know‐how, and three items (Item 22–24) covered the domain of personal protective gear and mouthguard use. Inclusion criteria to participate in the study were, to be a professional ski and/or snowboard instructor, work experience as ski and/or snowboard instructor of one season or more, teaching in Austria, Germany, or Switzerland, and informed consent to participate in the study. Using the questionnaire, a total of 603 professional ski and snowboard instructors fulfilling the eligibility criteria were interviewed between December 2019 and May 2020. Five hundred fifty‐two instructors were interviewed in the skiing areas where they worked, before or after their lessons. Fifty‐one Swiss instructors were interviewed by phone owing to the COVID‐19 pandemic. The instructors interviewed worked in one of the five biggest skiing areas of their respective countries. All interviews were conducted by the same investigator (G. U.). Data were sampled in a Microsoft Excel spreadsheet (Microsoft Corporation, Version 16.41) and further transferred into the statistical program R version 3.5.1 (version 3.5.3., R Foundation for Statistical Computing). Categorical variables were described by indicating the number and percentage, while continuous parameters like age were described by the mean and standard deviation (SD). Either Fisher's exact tests and *χ* ^2^ tests or Wilcoxon rank‐sum and Kruskal–Wallis tests were performed to detect significant differences between given groups. To evaluate potential parameters that affect the occurrence of injuries, logistic regression models were performed (injury: yes vs. no). The resulting estimates were odds ratios (ORs) with the corresponding 95% confidence interval (CI) and *p*‐value. In advance, it was tested for potential interactions in order to perform a separate regression analysis. The various parameters were stepwise analyzed in order to prevent confounding effects. In addition, linear regression analysis was performed to describe chosen effect sizes (e.g., age dependency in the case of injuries). Adjustment for multiple comparisons was omitted owing to the descriptive nature of the study. A *p *< .05 was considered as significant (two‐sided).

## 3 RESULTS

**Figure 1:**
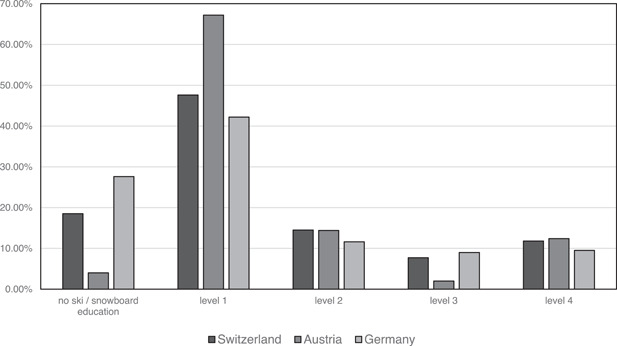
Comparison of the education statuses of the ski and snowboard instructors in Switzerland, Austria, and Germany in percent of all instructors who were interviewed. Every snow sport level consists of several days to weeks lasting practical training with ensuing practical and theoretical exam

The data set comprised data from 603 questionnaire‐based interviews with ski and snowboard instructors. The mean age of the interviewees was 27.8 (SD = 11). Each country—Austria, Germany, and Switzerland—was represented with an equal number of instructors (*n* = 201). Two hundred twenty‐three (37.5%) females and 378 (62.7%) males participated in the study, with no difference in the sex ratio across the three countries. One hundred thirty (21.6%) instructors taught both skiing and snowboarding, 416 (69%) taught exclusively skiing, and 57 (9.5%) taught exclusively snowboarding. Figure 1 shows the distribution of the different snow sport education levels in Switzerland, Austria, and Germany.

**Figure 2:**
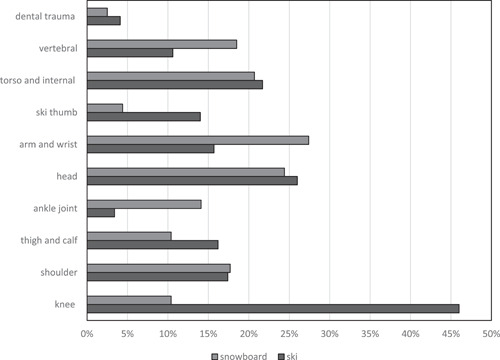
Comparison of types of ski and snowboard injuries in percent of total injuries that occurred

Two hundred and seventy‐seven (45.9%) of instructors reported no past injury while skiing or snowboarding. One hundred and ninety‐one (31.7%) reported a skiing‐related injury, 44 (7.3%) reported both skiing and snowboarding‐related injuries, and 91 (15.1%) reported a snowboarding‐related injury. Snowboarding and skiing showed no significant differences in terms of the rate of injuries (*p* = .0952). Males had a 2.7‐fold higher probability of incurring a skiing‐related injury compared with females (OR: 2.7; 95% CI [1.2, 5.9]; *p* = .015). The chance of sustaining a snowboard‐related injury was significantly lower for males compared with females (OR: 0.5; 95% CI [0.30, 0.96]; *p* = .036). Accidents resulting in injuries occurred most frequently on the slope (*n* = 233, 54.8%), followed by backcountry (*n* = 109, 25.6%), snow park (*n* = 63, 14.8%), at or on the lift (*n* = 11, 2.6%), and other (*n* = 9, 2.1%). Figure 2 shows the different injuries comparing ski and snowboard (for better comparability, all injuries were subdivided in 10 main groups).

**Figure 3:**
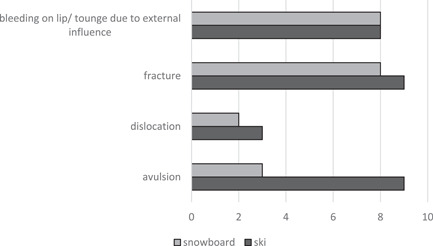
Ski and snowboard instructors' dental injuries in total

The likelihood for ski instructors of an injury in the backcountry tended to be higher than on the slope, yet not enough to be statistically significant (*p* = .057). The likelihood of an injury was three times lower for backcountry snowboarding compared with snowboarding on the slope (OR: 3.37; 95% CI [0.17, 0.18]; *p* = .012). Instructors reported the following causes for injury: 251 (64.2%) individual accidents, 58 (14.8%) incidents in the snow park, 42 (10.7%) collisions with other skier/snowboarder, 29 (7.4%) collisions with an obstacle, 11 (2.8%) other (technical problem with equipment, avalanche). The frequency of injuries to the following body parts showed no significant difference between ski and snowboard instructors: shoulder, thigh and calf, head, torso, and internal organs. Ski instructors incurred knee and ulnar collateral ligament injuries more frequently than snowboard instructors (*p* < .0001 and *p* = .028, respectively). Injuries of the ankle joint, arm and wrist, and vertebral column were more frequently reported by snowboard instructors compared with ski instructors (*p* = .0001, *p* = .0068, and *p* = .0324, respectively). Five hundred sixty‐three (93.4%) instructors gave an account of no dental injury related to skiing or snowboarding. Dental injuries related to skiing and snowboarding were reported by 25 (4.1%) and 15 (2.5%) instructors, respectively. Figure 3 shows the distribution of dental injuries among ski and snowboard instructors.

Skiing‐related dental injuries were more frequent among Swiss instructors compared with their Austrian and German colleagues (*p* ≤ .0025). Educational level had no impact on the frequency of dental injuries. 58.8% of instructors who sustained a snow sports‐related dental injury reported that they still feel or see the consequence of the dental injury. Five hundred and forty‐one (89.7%) of instructors reported that a dental injury never occurred to a student of theirs during the lesson. A student incurring a dental injury related to skiing and snowboarding was witnessed by 54 (9%) and 8 (1.3%) instructors, respectively.

**Figure 4:**
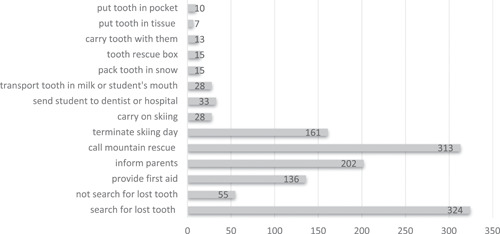
Instructors' reactions in a case of student's tooth avulsion with multiple answers possible. An open‐ended question

Figure 4 shows the answers ski and snowboard instructors gave to the question of what they would do in case of a tooth avulsion.

If one of their students sustained an avulsion injury, instructors from Austria reported more frequently that they would send the student to a dentist compared with instructors from Germany and Switzerland (*p* ≤ .012). Three hundred twenty‐seven (54.2%) reported that they thought it is possible for a dentist to replant an avulsed tooth whereas 276 (45.8%) deemed this impossible. Twenty‐three (3.8%) instructors reported that the management of dental injuries was part of their ski or snowboard education. The dental education took up less than an hour of their education and training. In comparison with German (*n* = 122, 60.1%) and Swiss instructors (*n* = 166, 82.6%), instructors in Austria (*n* = 194, 96.5%) reported significantly more frequently that they carry first aid material (*p* ≤ .001). First aid material was more frequently part of the equipment of instructors teaching skiing or skiing and snowboarding compared with instructors who teach exclusively snowboarding (*p* = .015). No difference between genders was observed in this regard. There was no difference among the answers between the age groups ≤25‐year‐old and >25‐year‐old instructors regarding whether or not an avulsed tooth can be reimplanted. Table 1 shows instructors first aid material in percent.

**Table 1:** Ski and snowboard instructors' first aid equipment in percent with multiple answers possible

First aid kit	79.9%
Different single materials	4.6%
Emergency blanket	4.6%
Wound dressing	3%
Adhesive plaster	2.7%
Compresses	1.8%
Analgetics	0.8%
Scissors	0.2%
Tooth rescue box	0%
None	16.1%

Sixty‐one (10.1%) of instructors were familiar with the tooth rescue box. Familiarity with the tooth rescue box was more common among German (*n* = 18, 9.0%) and Swiss instructors (*n* = 30, 14.9%) compared with Austrian instructors (*n* = 13, 6.4%), albeit not statistically significant (*p* ≤ .329). There was no statistically significant difference in knowledge of the tooth rescue box between the age groups ≤25 years old and >25 years old (*p* = .779). The 59 instructors familiar with the tooth rescue box reported to have gained their knowledge of the tooth rescue box in the following setting: in private (*n* = 29, 49.2%), in a first aid course (*n* = 16, 27.1%), during their ski/snowboard education (*n* = 5, 8.5%), in connection with their own kids (*n* = 3, 5.1%), or other (*n* = 6, 10.2%). Out of 603 instructors, 17 (2.8%) reported to wear no personal protective gear. Five hundred seventy‐six (95.6%) reported to wear a helmet while privately skiing or snowboarding. Men chose more frequently to refrain from wearing a helmet than females (*p* = .0139) and younger instructors (≤25) wore helmets more frequently than older instructors (>25) (*p* = .039). Two hundred and ninety‐five (48.9%) reported to wear a back protection, with no differences between genders and countries. Of those instructors who wore back protection, significantly more were younger than 25 years (*p* < .001). Back protection was more common among snowboard instructors than ski instructors (*p* = .0273). Of the 187 instructors teaching snowboarding, 6 (3.2%) reported to wear wrist protectors. Mouthguard use while privately skiing or snowboarding was reported by 21 (3.5%) of the instructors surveyed, with no difference between genders and no significant difference between older (age > 25) and younger (age ≤ 25) instructors (*p* = .412). Out of the 21 instructors who stated to wear a mouthguard, only three said to wear it generally while skiing/snowboarding, 12 reported wearing it in snow parks, three when backcountry skiing/snowboarding, four reported wearing it in races.

## 4 DISCUSSION

Based on survey data from 603 ski and snowboard instructors, this study indicates that over half of the instructors (54.1%) had sustained a snow sports‐related injury in the past. Dental injuries related to skiing and snowboarding among snow sports instructors were rare, with a reported frequency of 4.1% and 2.5%, respectively. The helmet usage rate of professional instructors was high (95.6%), whereas mouthguard usage was uncommon (3.5%). Professional snow sports instructors' know‐how in dental first aid was limited: close to half of instructors (45.8%) had no knowledge of the possibility of replanting avulsed teeth and only 1 in 10 instructors was familiar with the tooth rescue box.

In contrast to a previous study (Flørenes et al., 2009), the present investigation found no significant difference in the rate of injuries related to skiing and snowboarding. This may be due to fact that the present study included exclusively data from expert skiers and snowboarders, whose risk for injury is lower compared with beginners (Flørenes et al., 2009). The general injury rate of male skiers surveyed in the study was almost three times higher than that of female ski instructors, with previous studies suggesting more risk‐taking behavior among males as the underlying reason for this discrepancy (Flørenes et al., 2009; Willick et al., 2019). Conversely, in line with a previous study (Dickson & Terwiel, 2021), the injury rate of female snowboard instructors was found to be higher than that of their male colleagues. The reason for this finding remains elusive. Regarding the terrain where surveyed instructors sustained an injury, significant differences were observed between skiers and snowboarders. While backcountry skiing was associated with an increased risk of injury compared with skiing on the slope, backcountry snowboarding entailed fewer injury risks compared with snowboarding on the slope. Varying snow conditions provide a likely explanation for this finding: on backcountry terrain, deep snow abounds while the snow on the trail of a slope is usually groomed. Skiing in deep snow may exert stronger torsional forces on the lower extremities and consequently increase the risk of sprain injuries to the knee and leg. Snowboarders, more prone to upper extremity injuries than skiers, carry a higher injury risk on groomed, compacted slopes, where impact forces tend to be higher (Langran & Selvaraj, 2002).

Previous studies reported that knee injuries are the most common type of injury sustained while skiing with reports ranging from 27% to 41% of all injuries (Burtscher et al., 2008; Johnson et al., 2008; Patrick et al., 2015). The present investigation provides corroborating evidence for this finding. Knee injuries accounted for 47% of injuries of ski instructors. By contrast, arm and wrist injuries, accounting for 27.4% of injuries, were the most frequent type of injury among snowboard instructors. 6.6% of ski and snowboard instructors reported a snow sports‐related dental injury or a lip or tongue injury. A previous study, comprising survey data from 500 amateur skiers and snowboarders, found 2.2% of interviewees with a dental injury sustained while skiing or snowboarding (Innerhofer et al., 2013). The increased rate of dental injuries among snow sports instructors may be due to their spending considerably more time doing sports or more risk‐taking behavior or both. Yet, compared with other sports, this rate of dental injuries sustained by snow sports instructors is low. For instance, the rate of dental injuries in martial arts and handball athletes is 32% and 37%, respectively (Ferrari et al., 2002).

Among snow sports students, the occurrence of dental injuries showed no difference between genders. This is in accordance with previous studies that found no difference in risk‐taking behavior between boys and girls (Ginsburg & Miller, 1982). Moreover, the present investigation found that children under the age of 14 are more susceptible to dental injuries, which is consistent with previous research that has also shown that younger children are at an increased risk for accidents (Laver et al., 2017).

Professional snow sports instructors' helmet usage rate of 95.6%—while skiing in private—was high with an even higher proportion in instructors under the age of 25 years. Younger instructors also wore back protective gear more than older instructors. A previous study demonstrated that protective gear usage increases with athletic experience (Innerhofer et al., 2013), which is concordant with the high prevalence of helmet usage among the expert skiers and snowboarders surveyed in the present investigation. Increased helmet use, advances in equipment and binding systems, and advances in slope grooming have led to a decrease in the number of skiing‐related injuries over the past years (Johnson et al., 2008; Willick et al., 2019). In comparison with amateur skiers and snowboarders (Innerhofer et al., 2013), the rate of mouthguard use (3.5%) among snow sports instructors was higher. However, the present study indicates that snow sports instructors choose to wear a mouthguard only for selected occasions such as backcountry skiing or snowboarding, or snow park use, where the perceived risk for dental injury is higher. Wrist protection usage was rare among snowboard instructors. The reason for this may be twofold: First, wrist protectors cause some discomfort (Michel et al., 2013) and, second, wrist protection gear usage may increase the chance of injuries to the elbow and shoulder joint (Bianchi et al., 2012; Chow et al., 1996; Düwell, 2015).

The present study found significant gaps in snow sports instructors' knowledge of dental first aid. Half of the instructors participating in the survey reported that they would search for an avulsed permanent tooth and only 54.2% knew that an avulsed tooth may be replanted. To prevent desiccation of periodontal ligament cells, it is crucial to keep the extra‐alveolar dry time as short as possible. In dry conditions, necrosis of periodontal ligament cells occurs within 16–30 min: any prolonged duration of dry storage of an avulsed tooth, therefore, dramatically decreases the chance of periodontal healing after replantation (De Brier et al., 2020). Though immediate replantation is the ideal treatment for an avulsed tooth (Poi et al., 2013), current guidelines advise against laypeople undertaking the replantation. Tooth rescue boxes contain an ideal storage medium for avulsed teeth. However, of the instructors surveyed, only 10.1% were familiar with the tooth rescue box and none of them had a tooth rescue box in their first aid equipment. If a tooth rescue box is not at hand, milk—usually readily available—can be used as a storage medium for an avulsed tooth as it can preserve cell viability for up to 6 h (Blomlöf et al., 1983; Pearson et al., 2003). Yet only 4.6% of instructors would transport an avulsed tooth in milk or the injured student's mouth. Considering how essential it is to deliver adequate first aid in case of tooth avulsion injuries, the present study reveals that concerted efforts are required to enhance dental first aid know‐how in snow sports communities. Ski and snowboard instructors are an important target audience, which is already well trained in general first aid. Measures to improve dental first aid—from basic education to the propagation of tooth rescue boxes—are easy to adopt and implement but collaboration with relevant stakeholders is needed to effectively disseminate such measures.

In conclusion, the study shows that use of protective gear while privately skiing/snowboarding was high for those expert skiers. Even though the frequency of oral injuries was lower compared with other sports, dental first aid know‐how was insufficient among instructors and ought to be enhanced in snow sports communities.

## CONFLICT OF INTERESTS

The authors declare that there are no conflict of interests.

## AUTHOR CONTRIBUTIONS

*Conceptualization*: Andreas Filippi and Greta Unzeitig. *Methodology*: Andreas Filippi and Greta Unzeitig. *Validation*: Andreas Filippi and Greta Unzeitig. *Investigation*: Greta Unzeitig. *Formal analysis*: Andreas Filippi, Greta Unzeitig, and Florin Eggmann. *Data curation*: Greta Unzeitig. *Writing–original draft preparation*: Greta Unzeitig and Florin Eggmann. *Writing–review and editing*: Andreas Filippi, Greta Unzeitig, and Florin Eggmann. *Visualization*: Greta Unzeitig. *Supervision*: Andreas Filippi. *Project administration*: Andreas Filippi and Greta Unzeitig. *Funding acquisition*: Andreas Filippi.

## Data Availability

The data that support the findings of this study are available from the corresponding author upon reasonable request.

## 1

Questionnaire

**Table:**

1. Age^a^
2. Gender (female, male, else)
3. Current workplace? (Austria, Germany, Switzerland)
4. Do you teach skiing, snowboarding, or both?
5. Which is the highest educational ski/snowboard level, you have archived? (no education, Level 1, Level 2, Level 3, Level 4 [state certified ski/snowboard instructor])
6. Have you ever sustained an injury while skiing or snowboarding? (no; yes, snowboarding; yes, skiing; yes, both)
7. On what kind of terrain did you sustain the injury? (slope, backcountry, snow park, at or on a lift, other)
8. What was the cause? (individual accident, collision with other skier/snowboarder, collision with obstacle, snow park, other)
9. What kind of injury did you incur?^a^
10. Have you ever sustained a dental injury while skiing/snowboarding? (yes, skiing; yes, snowboarding; both; no)
11. What kind of dental injury? (avulsion, dislocation, fracture, bleeding on lip/tongue due to external influence/bite)
12. What happened afterward?^a^
13. Do you still feel/see the consequences of the injury? (yes/no)
14. Has any of your students ever sustained a dental injury in your lesson? (yes, while snowboarding, yes, while skiing, no)
15. What would you do if one of your students suffered a tooth avulsion on the slope?^a^
16. Do you think a dentist could replant an avulsed tooth? (yes, no)
17. Did you learn anything about dental injuries in your ski/snowboard education? (yes, no)
18. If yes, how much time was spent on education on dental injuries? (up to 1 h, 1–2 h, half a day, more)
19. Do you carry first aid material with you in your lessons? If yes, what? (yes, single things like emergency blanket, dressing, plaster, compresses, painkillers, tooth rescue box, scissors, gloves; whole first aid set; no, nothing)
20. Are you familiar with the tooth rescue box? (yes/no)
21. Where do you know the tooth rescue box from? (private, ski/snowboard education, first aid course, own kids, other^a^)
22. Do you wear a mouthguard while privately skiing/snowboarding)? (yes, no)
23. If yes, during which activities? (generally, snow park, backcountry)
24. Do you wear any other protective gear while privately skiing/snowboarding? (wrist protection, back protection, helmet, other^a^, no)

a Were open‐ended questions.
